# P15 A Juvenile idiopathic arthritis mimic

**DOI:** 10.1093/rap/rkab068.014

**Published:** 2021-10-19

**Authors:** Charlene Foley, Kate Armon, Peter Bale

**Affiliations:** Addenbrooke's Hospital, Cambridge, United Kingdom

## Abstract

**Case report - Introduction:**

Juvenile idiopathic arthritis (JIA) is a diagnosis of exclusion. In a paediatric rheumatology clinic, children can present with signs and symptoms that don’t quite fit under the umbrella of JIA. When these cases present it is important that we take a thorough history and examination, and investigate as appropriate, with continual assessment to check progress and review response to treatment. This case highlights the importance of this practise, in turn enabling the correct diagnosis to be reached. This allows for more appropriate treatment choices and more specific counselling for child and family with regards to the condition and expected prognosis.

**Case report - Case description:**

A 7-year-old girl was referred for paediatric rheumatology review with a 3-week history of inability to close her hands properly, and pain and swelling in hands and feet. There had been no preceding illness, trauma, or tick bites. There was no history of associated fever, rash, mouth ulcers or eye symptoms. She complained of central abdominal pain with associated constipation. Her appetite and weight were stable. No significant PMH. FHx brother - JIA.

On assessment, systems exam was unremarkable. Her skin was soft with no erythema. It did not appear thickened or shiny, but there was bilateral pitting oedema to just below both knees. On musculoskeletal assessment there was notable dorsal swelling of both hands, with significant restriction of both wrists. There was pain, swelling and restriction of the fingers, elbows, knees, ankles and toes. Her neck and shoulder movements were also restricted.

**Limited summary of investigation:**

- WBC 18.5, Eosinophils 12.77
- Albumin 24 (NR 30-50 g/L)
- ESR 2-7, CRP 6-12
- Infection screen / Autoimmune / Scleroderma-Ab-profile / Myositis-Ab-profile NAD
- X-rays Hands/Feet – NAD
- Abdominal USS NAD
- Urine - culture and Alb/Cr ratio NAD
- Stool culture / parasites / ova – NAD
- Ophthalmology review NAD

An USS was performed of affected joints which showed poly-articular synovitis and tenosynovitis. There was no documented fasciitis. She was treated with three-days IV methylprednisolone (30mg/kg) and discharged on a weaning course of prednisolone.

She was reviewed 3-weeks later. She was functioning better but reported on-going abdominal pain and that her arms looked thicker. The skin on her arms appeared thickened. She was unable to make a claw or fully extend fingers. Her wrists were restricted with visible wrist flexor tendons. Her pitting oedema had improved. Her inflammatory markers were normal. She proceeded to have an MRI of her right hand/wrist which confirmed a diagnosis of eosinophilic fasciitis and tenosynovitis.

**Case report - Discussion:**

Following initial assessment, this 7-year-old girl was found to have significant inflammatory arthritis and tenosynovitis, abdominal pain, eosinophilia and hypo-albuminaemia. However, the clinical picture was not felt to be typical for JIA. The differential diagnosis at the time was that this could be an evolving connective tissue disease; however, the autoimmune screen was negative. Also, initially there were no abnormal skin or fascial features seen on imaging, and no other organ dysfunction or features pointing towards a specific condition.

Once infection and risk of malignancy was excluded in consultation with the infectious disease and haematology teams, it was agreed that treatment was required. Pulse methylprednisolone was agreed as an appropriate first choice with a plan to review response on weaning oral steroids.

When the little girl returned for review, the clinical picture had progressed to include non-tender, tightness and thickening of the forearms, with ongoing restriction in multiple joints and abdominal pain. In view of the ongoing abdominal pain, eosinophilia, low albumin and rising ALT she was discussed with the gastroenterology team who repeated an abdominal USS and performed an OGD. The USS showed a slightly enlarged spleen. The OGD was macroscopically normal. Microscopically there were some submucosal duodenal foamy macrophages, significance uncertain.

The team remained suspicious that this was an eosinophilic driven inflammatory condition with musculoskeletal findings, and so an MRI of the right hand and wrist was requested. This showed an abnormal rim of high signal around the tendons and along the fascial planes of the forearm in both extensor and flexor compartments, consistent with a diagnosis of eosinophilic fasciitis. A skin and muscle biopsy were obtained which confirmed chronic inflammation and fibrosis in the fascia. These features were consistent with the suggested radiological diagnosis of eosinophilic fasciitis. She was commenced on subcutaneous methotrexate.

**Case report - Key learning points:**

Eosinophilic faciitis (EF), also known as Shulman syndrome after the physician who, in 1974 was the first to report on the disorder in the medical literature, is a rare disorder. It is characterised by inflammatory infiltrate in the fascia consisting of lymphocytes, macrophages and plasma cells, with eosinophils sometimes present. The fascia is thickened 2- to 15- fold; the dermis and epidermis can be unaffected.

The condition can precede, coexist or follow localised scleroderma. Clinically it can present as painful swelling with progressive induration and thickening of the skin – “peau d’orange” appearance. In paediatrics, as was the case in this little girl, skin findings are minimal or absent at presentation. Paediatric EF tends to involve extremities, often the hands and feet.

There is a paucity of data for paediatric EF. In childhood onset EF there is a higher frequency of joint involvement. However, many can develop persistent cutaneous fibrosis and permanent disability. Over 30% of children also present with visceral involvement, such as mesenteric lymphadenopathy, hepatosplenomegaly and pericardial effusion.

Early recognition of the condition and timely initiation of treatment improves likelihood of good response to treatment. Risk factors for persistent fibrosis include extensive disease (3—4 extremities and trunk involvement) and/or younger age at onset.

There are no standardised guidelines for treatment of childhood-onset EF. However, given that children have more severe, rapidly progressive articular involvement, the expert view is that initial treatment should include combination therapy i.e., corticosteroids and methotrexate. If this treatment fails, the literature suggests instigation of alternative DMARDS, biologics or JAK inhibitors.

Summary learning/discussion points include;

- EF is Rare, with paucity of paediatric data
- Comprehensive work up required
- Early, aggressive treatment important to achieve best outcomes
- Thorough skin examination important to monitor for localised scleroderma
- No evidence-based treatment pathway available in paediatric EF

